# Supplementary figures and images for: Structure of Dunaliella photosystem II reveals conformational flexibility of stacked and unstacked supercomplexes
Source: eLife. 2023 Feb 17;12:e81150. doi: 10.7554/eLife.81150 (PMC9949808; doi:10.7554/eLife.81150)

## Slide 1
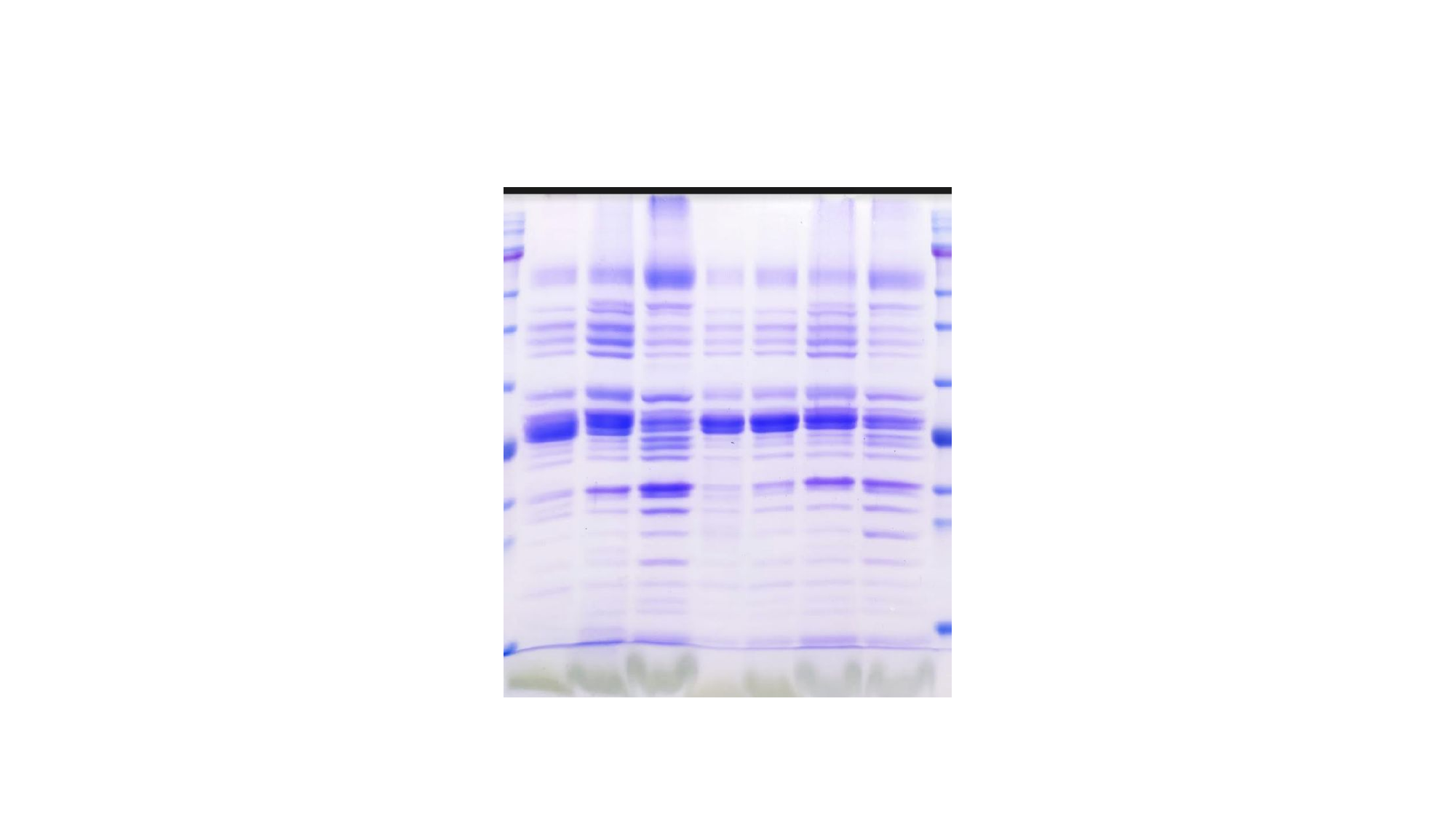

## Slide 2
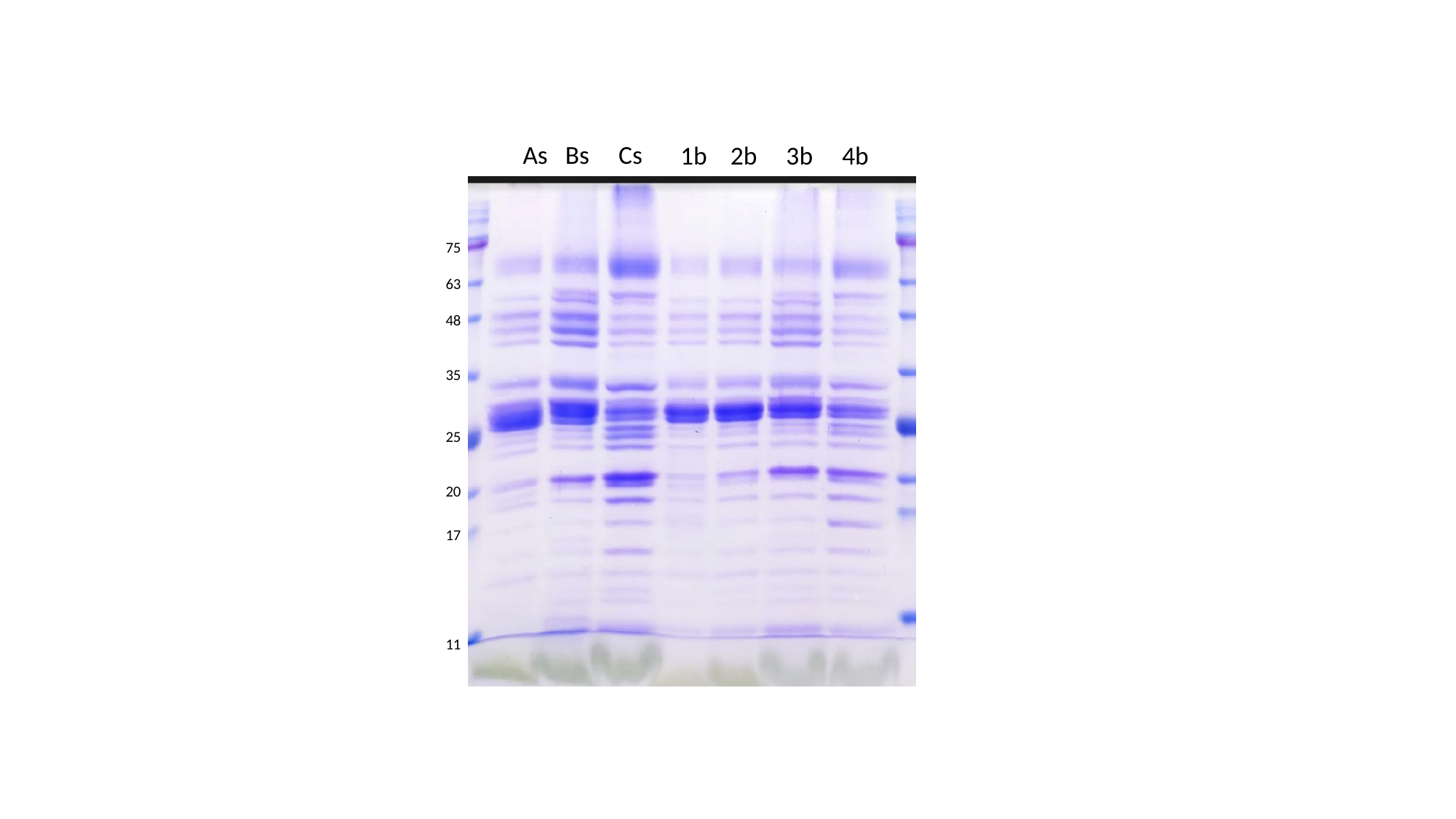

As Bs Cs
1b 2b 3b 4b
75
63
48
35
25
20
17
11

Supplement: Figure 1—figure supplement 9—source data 1. [file elife-81150-fig1-figsupp9-data1.pptx]
