# Supplementary material for: Structure of Dunaliella photosystem II reveals conformational flexibility of stacked and unstacked supercomplexes
Source: eLife. 2023 Feb 17;12:e81150. doi: 10.7554/eLife.81150 (PMC9949808; doi:10.7554/eLife.81150)
Supplement: Supplementary file 1. [file elife-81150-supp1.docx]

**Supplementary File 1.** **Cryo-EM data collection, refinement, and validation statistics.**

|  | C2S2_COMP_  (EMDB-13429)  (PDB 7PI0) | C2S2_STR_  (EMDB-13430)  (PDB 7PI5) | C2S  (EMDB-13548)  (PDB 7PNK) | Stacked C2S2_COMP_  (EMDB-13444)  (PDB 7PIN) | Stacked C2S2_STR_  (EMDB-13445) (PDB 7PIW) |
| --- | --- | --- | --- | --- | --- |
| **Data collection and processing** |  |  |  |  |  |
| Magnification | 130,000 | 130,000 | 130,000 | 130,000 | 130,000 |
| Voltage (kV) | 300 | 300 | 300 | 300 | 300 |
| Electron exposure (e^–^/Å^2^) | 51.81 | 51.81 | 51.81 | 51.81 | 51.81 |
| Defocus range (μm) | 0.8-1.9 | 0.8-1.9 | 0.8-1.9 | 0.8-1.9 | 0.8-1.9 |
| Pixel size (Å) | 0.64 | 0.64 | 0.64 | 0.64 | 0.64 |
| Symmetry imposed | C1 | C1 | C1 | C1 | C1 |
| Initial particle images (no.) | 401,467 | 401,467 | 401,467 | 401,467 | 401,467 |
| Final particle images (no.) | 39,357 | 23,014 | 21,066 | 9,567 | 14,307 |
| Map resolution (Å)  FSC threshold | 2.43  0.143 | 2.62  0.143 | 3.61  0.143 | 3.36  0.143 | 3.84  0.143 |
| Map resolution range (Å) | 2.0-4.0 | 2.0-4.0 | 3.0-7.0 | 3.2-8.0 | 3.2-8.0 |
|  |  |  |  |  |  |
| **Refinement** |  |  |  |  |  |
| Initial model used (PDB code) | 6KAC | 6KAC | 6KAC | 6KAC | 6KAC |
| Model resolution (Å)  FSC threshold | 2.70  0.143 | 2.70  0.143 | 2.70  0.143 | 2.70  0.143 | 2.70  0.143 |
| Model resolution range (Å) | 2.4-5.6 | 2.4-5.6 | 2.4-5.6 | 2.4-5.6 | 2.4-5.6 |
| Map sharpening *B* factor (Å^2^) | -59.66 | -41.14 | -64.65 | -58.56 | -73.16 |
| Model composition  Non-hydrogen atoms  Protein residues  Ligands | 77,465  7,442  344 | 76,299  7,455  343 | 60,673  6,142  250 | 152,602  14,846  722 | 151,508  14,859  703 |
| *B* factors (Å^2^)  Protein  Ligand  Water | 17.31-79.42  25.91-43.95  23.43-54.39 | 5.21-68.64  11.99-99.23  18.93-47.31 | 15.68-156.42  16.63-92.29  - | 16.33-192.00  20.05-206.46  - | 20.51-263.90  25.08-210.69  - |
| R.m.s. deviations  Bond lengths (Å)  Bond angles (°) | 0.005  0.953 | 0.007  1.596 | 0.005  1.513 | 0.005  1.632 | 0.009  1.681 |
| Validation  MolProbity score  Clashscore  Poor rotamers (%) | 1.72  8.84  0.0 | 1.93  10.60  0.05 | 1.93  11.86  0.08 | 1.96  12.29  0.04 | 2.10  14.68  0.03 |
| Ramachandran plot  Favored (%)  Allowed (%)  Disallowed (%) | 96.33  3.60  0.07 | 94.35  5.24  0.41 | 95.06  4.79  0.15 | 94.73  5.11  0.16 | 93.53  6.19  0.27 |
