# Supplementary material for: Structure of Dunaliella photosystem II reveals conformational flexibility of stacked and unstacked supercomplexes
Source: eLife. 2023 Feb 17;12:e81150. doi: 10.7554/eLife.81150 (PMC9949808; doi:10.7554/eLife.81150)
Supplement: Supplementary file 2. [file elife-81150-supp2.docx]

**Supplementary File 2: CP29 chlorophyll composition determined in previous work.**

| Chl #  PDBID | PSII type | Res  (Å) | 601^*a^ | 602 | 603 | 604 | 606 | 607 | 608 | 609 | 610 | 611 | 612 | 613 | 614 | 615* | 616^a^ |
| --- | --- | --- | --- | --- | --- | --- | --- | --- | --- | --- | --- | --- | --- | --- | --- | --- | --- |
| plants |  |  |  |  |  |  |  |  |  |  |  |  |  |  |  |  |  |
| 3PL9 | Monomer | 2.8 | - | a | a | a | b | b | b | a | a | a | a | a | b | a | - |
| 5XNM | C2S2M2 | 3.2 | a | a | a | a | b | b | b | a | a | a | a | a | a | - | a |
| 5XNL | C2S2M2 | 2.7 | a | a | a | a | b | b | b | a | a | a | a | a | b | - | a |
| 7OUI | C2S2M2 | 2.8 | a | a | a | a | b | b | b | a | a | a | a | a | - | a | a |
| 3JCU | C2S2 | 3.2 | a | a | a | a | b | b | b | a | a | a | a | a | - | - | a |
| 6YP7 | C2S2 | 3.8 | - | a | a | a | b | b | b | a | a | a^b^ | - | a | - | - | - |
| alga |  |  |  |  |  |  |  |  |  |  |  |  |  |  |  |  |  |
| 6KAD | C2S2M2L2 | 3.4 | a | a | a | a | b | b | b | a | a | a | a | a | - | - | a |
| 6KAF | C2S2M2L2 | 3.7 | a | a | a | a | b | b | b | a | a | a | a | a | - |  | a |
| 6KAC | C2S2 | 2.7 | - | a | a | a | b | b | b | a | a | - | - | - | - | - | - |
| 7PI0 | C2S2_comp_ | 2.4 | - | a | a | a | b | b | b | a | a | - | a | - | - | - | - |
| 7PI5 | C2S_str_ | 2.8 | - | a | a | a | b | b | b | a | a | a | a | a | - | - | - |

^*^ - These chlorophylls occupy mutually exclusive positions.

^a^ – Chl numbering from PDBID: 5XNM

^b^ – large positional shift (chain R, residue 310 in 6YP7).
