# Supplementary material for: Structure of Dunaliella photosystem II reveals conformational flexibility of stacked and unstacked supercomplexes
Source: eLife. 2023 Feb 17;12:e81150. doi: 10.7554/eLife.81150 (PMC9949808; doi:10.7554/eLife.81150)
Supplement: Supplementary file 3. — Shift in chlorophyll position between states 1 and 10 in the first three PCs of the compact (Comp) and stretched (Str) unstacked PSII. Distances are in Å. The average shift and SD are presented for each component. [file elife-81150-supp3.docx]

**Supplementary File 3. Changes in location of CP29 chlorophylls in the compact and stretched conformations PCs.** Shift in chlorophyll position between state 1 and 10 in the first three PCs of the compact (Comp) and stretched (Str) unstacked PSII. Distances are in Å. The average shift and standard deviation are presented for each component.

| **CP29 Chl** | **602** | **603** | **604** | **606** | **607** | **608** | **609** | **610** | **612** | **Average shift** | **Stdev** |
| --- | --- | --- | --- | --- | --- | --- | --- | --- | --- | --- | --- |
| **Comp PC1** | 4.8 | 3.7 | 2.2 | 2 | 3.3 | 1.3 | 2.4 | 2.2 | 3.5 | **2.8** | **1.1** |
| **Comp PC2** | 0.5 | 0.4 | 0.7 | 0.3 | 0.3 | 0.6 | 0.3 | 0.7 | 0.5 | **0.5** | **0.2** |
| **Comp PC3** | 6 | 4.9 | 5.7 | 5 | 4.6 | 5.4 | 4.6 | 6.4 | 7.3 | **5.5** | **0.9** |
|  |  |  |  |  |  |  |  |  |  |  |  |
| **Str PC1** | 1.1 | 0.8 | 3.3 | 3.2 | 3 | 2.3 | 0.9 | 2.6 | 2.8 | **2.2** | **1.0** |
| **Str PC2** | 5.7 | 5.3 | 6 | 5.3 | 5.7 | 4.6 | 4.6 | 5.5 | 6.7 | **5.5** | **0.7** |
| **Str PC3** | 3.1 | 2.6 | 3.4 | 3 | 2.9 | 2.9 | 2.5 | 3.5 | 4.1 | **3.1** | **0.5** |
